# Using video-reflexive ethnography and simulation-based education to explore patient management and error recognition by pre-registration physiotherapists

**DOI:** 10.1186/s41077-016-0010-5

**Published:** 2016-03-22

**Authors:** Suzanne Gough, Abebaw Mengistu Yohannes, Janice Murray

**Affiliations:** Department of Health Professions, Manchester Metropolitan University, Manchester, UK

**Keywords:** Error recognition, Deterioration, Physiotherapy, Simulation-based education, Video-reflexive ethnography

## Abstract

**Background:**

Upon graduation, physiotherapists are required to manage clinical caseloads involving deteriorating patients with complex conditions. In particular, emergency on-call physiotherapists are required to provide respiratory/cardio-respiratory/cardiothoracic physiotherapy, out of normal working hours, without senior physiotherapist support. To optimise patient safety, physiotherapists are required to function within complex clinical environments, drawing on their knowledge and skills (technical and non-technical), maintaining situational awareness and filtering unwanted stimuli from the environment. Prior to this study, the extent to which final-year physiotherapy students were able to manage an acutely deteriorating patient in a simulation context and recognise errors in their own practice was unknown.

**Methods:**

A focused video-reflexive ethnography study was undertaken to explore behaviours, error recognition abilities and personal experiences of 21 final-year (pre-registration) physiotherapy students from one higher education institution. Social constructivism and complexity theoretical perspectives informed the methodological design of the study. Video and thematic analysis of 12 simulation scenarios and video-reflexive interviews were undertaken.

**Results:**

Participants worked within the professional standards of physiotherapy practice expected of entry-level physiotherapists. Students reflected appropriate responses to their own and others’ actions in the midst of uncertainty of the situation and physiological disturbances that unfolded during the scenario. However, they demonstrated a limited independent ability to recognise errors. Latent errors, active failures, error-producing factors and a series of effective defences to mitigate errors were identified through video analysis. Perceived influential factors affecting student performance within the scenario were attributed to aspects of academic and placement learning and the completion of a voluntary acute illness management course. The perceived value of the simulation scenario was enhanced by the opportunity to review their own simulation video with realism afforded by the scenario design.

**Conclusions:**

This study presents a unique insight into the experiences, skills, attitudes, behaviours and error recognition abilities of pre-registration physiotherapy students managing an acutely deteriorating patient in a simulation context. Findings of this research provide valuable insights to inform future research regarding physiotherapy practice, integration of educational methods to augment patient safety awareness and participant-led innovations in safe healthcare practice.

**Electronic supplementary material:**

The online version of this article (doi:10.1186/s41077-016-0010-5) contains supplementary material, which is available to authorized users.

## Background

In 2009, the Chief Medical Officer’s report to the UK’s Department of Health [1] stated that simulation offers an important route to safer care for patients and needs to be more fully integrated into healthcare education. Over the last two decades, evidence has demonstrated the benefits of simulation-based education (SBE) in healthcare [2–6]. There is now strong evidence that SBE improves learning outcomes [7, 8] and clinical practice [6, 9, 10], improving confidence, competency and clinical decision-making [11]. The integration of SBE within training and education has been shown to be cost-effective and associated with significant health-related cost savings [12]. The drive to improve patient safety has drawn upon SBE to support the quality to organisational priorities and address healthcare system failures [13], reduce adverse events [14] and reduce in-hospital infection rates [15]. It is also recognised that optimal advantages of SBE are realised when the interventions are appropriately designed and evaluated [16–17]. Negative SBE learning experiences may arise due to ill-designed scenarios [18], particularly when a learner is cognitively overloaded [19–21]; inappropriate levels of fidelity and realism; inadequately trained simulation faculty; ineffective facilitation techniques; and lack of or ineffective feedback [16, 18, 22, 23]. The barriers to implementing SBE include the complexity of scheduling simulation experiences within academic curricula and in other healthcare training environments, financial and time costs associated with the need for high staff to learner ratio, lack of available equipment to ensure equity of provision, lack of technical support and lack of funding for simulation resources [24–28].

SBE is not a new teaching modality within the physiotherapy profession. Clinical skill development (experienced during practice placements) on ‘real’ patients is deemed an essential component in the development of professional skills and has been used within physiotherapy education since the inception of the profession in 1895 [28, 29]. SBE modalities reported in physiotherapy literature included role-plays involving simulated patients (peers, actors or volunteers trained to portray the role of a patient), paper vignettes, use of part-task trainers, haptic simulators, virtual reality simulators and computerised full-body manikins [22, 24–38]. The extent to which SBE has been embedded within pre-registration physiotherapy curricula was outlined in a national survey in Australia [30]. A review of the UK provision and use of simulation was commissioned by the Department of Health in 2009. This review highlighted varied provisions and uses across the UK in medical, nursing and midwifery, allied health professional and clinical psychology education and training [22]. However, only one reference was made to the use of SBE within a single physiotherapy programme and no reported use within postgraduate physiotherapy training provided by National Health Service Trusts. Gough et al. [28] reported that SBE was used to teach a wide variety of cardio-respiratory skills relevant to the acute respiratory and emergency on-call physiotherapy environments. National consistencies in adoption, availability, fidelity and accessibility have been similarly reported by both Australian and UK surveys [28, 30].

Jull et al. [30] reported the use of problem-based learning or case-based learning approaches (featuring lectures, tutorials, practical sessions, clinical education and simulation-based learning experiences). However, the report lacked specific detail of the use and application of each simulation medium (mode of delivery). Three formal pedagogies that underpin Australian physiotherapy curricula include a constructivist approach, computer-assisted learning approach and Blooms taxonomy, which have been reported by Jull et al. [30]. The physiotherapy literature reports the use of facilitator-led (instructional) methods within pre-registration and postgraduate physiotherapy education [28, 30]. No studies were identified that have involved student-led SBE in physiotherapy. SBE has been predominantly used in cardio-respiratory and musculoskeletal physiotherapy [31–35] and patient safety education [27]. Respiratory, musculoskeletal and neurological physiotherapy are an integral part of pre-registration physiotherapy education [36, 37]. Physiotherapy programmes are required to demonstrate compliance with all nine of the Chartered Society of Physiotherapy’s (CSP) learning and development principles [38], to prepare learners to the continually changing healthcare environment. In addition, programmes are required to incorporate the CSP’s physiotherapy framework: putting physiotherapy behaviours, values, knowledge and skills into practice [38]. Globally, physiotherapy students are required to complete 1000 h of placement-based education to prepare them for immediate clinical practice on graduation [30, 39].

The CSP recognised that newly qualified physiotherapists should be competent in respiratory care but will require further educational opportunities before becoming competent within the cardio-respiratory on-call context [39]. Concerns have been repeatedly raised regarding some physiotherapists’ abilities to deliver on-call respiratory physiotherapy in the UK [39–42]. The CSP published ‘*Emergency Respiratory On-call Working: Guidance for Physiotherapists*’ [39], in response to longstanding concerns regarding the delivery of physiotherapy care to patients who are at risk of deterioration (compromised respiratory function) outside of normal working hours (traditionally, 8.30 am–4.30 pm). Discrepancies in training within respiratory physiotherapy services have been identified by numerous UK surveys pertaining to respiratory care and on-call physiotherapy [41, 43, 44]. Roskell and Cross [45] described the complex interactions a respiratory physiotherapist undertakes to function effectively within their clinical environment. The importance of the physiotherapist’s non-technical skills has been recognised including the need to maintain situational awareness to efficiently function whilst optimally managing the patient [45]. A recent study has highlighted that significant disparities in treatment outcomes have been reported when paediatric patients are treated by non-respiratory on-call physiotherapists compared to specialist respiratory physiotherapists [42].

This article will focus on the following research questions:

1. To what extent are final-year pre-registration physiotherapy students able to independently manage an acutely deteriorating patient in a simulation context?
2. To what extent are final-year physiotherapy students able to independently recognise errors within a simulation-based learning experience?
3. Which elements of prior learning do pre-registration physiotherapy students perceive may influence their performance within a simulation-based learning experience?
4. What value do pre-registration physiotherapy students attribute to the cardio-respiratory simulation-based learning experience?

This article builds on previous research by providing unique insights into the experiences, skills, attitudes, behaviours and error recognition abilities of pre-registration physiotherapy students managing an acutely deteriorating patient in a simulation context. Prior to this study, pre-registration physiotherapy students’ acute illness management skills, error recognition abilities, concomitant influential factors affecting performance within physiotherapy simulation scenarios and the perceived value of SBE in physiotherapy education had not been explored.

## Methods

Prior to commencement of this study, university ethical approval was obtained (reference number 1102). This study follows an earlier study reporting a comprehensive examination of the use of SBE in cardio-respiratory physiotherapy education in the UK [28, 46]. Data was collected using survey methods. Findings from these surveys have been presented elsewhere [28, 46] and were used to develop the SBE resources presented in this paper. The current research study was informed by social constructivism [47, 48] and socio-material (complexity) theoretical perspectives [49–51]. Video-reflexive ethnography (VRE) methodology [52–54] was used to explore performance, behaviours and personal experiences of final-year (pre-registration) physiotherapy students from one UK higher education institution. Video-observation and focused, unedited video-reflexive interview methods were selected to capture multiple perspectives (approaches and understandings) and the complexity of managing an acutely deteriorating patient in a simulation context. A debrief was undertaken to resolve any erroneous events or discussions arising from the scenario or VRE interview. The VRE interview schedule was mapped to the research questions and explored achievement of the learning objectives, strengths and areas for improvement, and thus, these areas were not repeated in the debrief. The debrief ensured that the participants were aware of any errors or intervention that may impact on patient safety and discussed how they could be mitigated in the future, if these were not already addressed in the VRE interview.

### Participants

Twenty-seven final-year pre-registration physiotherapy students volunteered and consented to participate (34 % of the maximum available sample size of 85). Random allocation of participants to dates was not undertaken. Accommodation of participants’ preferred dates was undertaken to reduce the burden of participation and potentially the dropout rate. Despite allocating two students per simulation session according to participants’ preferences, three withdrew during the course of the study, three participated in the pilot and the remaining 21 completed the scenario and VRE interview reported in this article. Two doctoral students (from within the faculty) volunteered to undertake the role of the healthcare assistant in place of the withdrawn participants. The two volunteers both had prior experience of participating in SBE within the physiotherapy programme and were pre-briefed with the respective physiotherapy participants. In the three scenarios, the pre-registration physiotherapy participant was allocated to the role of the physiotherapist and the doctoral healthcare student was assigned to the healthcare assistant role (volunteers A and B). In the scenarios where a volunteer healthcare assistant (A or B) was involved, only the pre-registration physiotherapy students participated in the VRE interview. Thus, in the remaining nine scenarios, all participants were pre-registration physiotherapy students. All 21 pre-registration physiotherapy students participated in both the scenarios and VRE interviews.

### Data collection tools

The tools included video recording of the scenario and VRE interview. The VRE interview consisted of 21 specifically designed questions (available from the corresponding author), which aimed to promote self-reflection, whilst engaging in a critical discussion of themselves and their clinical practices [50]. The VRE interview schedule mapped directly to the research questions.

### Data collection

Table 1 illustrates the seven key elements that underpinned the simulation design, development and analysis of the study. An authentic scenario (Table 2) was purposely designed to replicate the complexity of an emergency on-call physiotherapy situation involving an acutely deteriorating cardio-respiratory in-patient. The scenario was based on the findings from the national surveys undertaken in phase 1 [28, 46] and an anonymised patient case study. Participants were randomly assigned to either the role of the physiotherapist or healthcare assistant (HCA) in the scenario and orientated to the simulation environment and its equipment before receiving the pre-brief. Immediately following the scenario, a VRE interview was conducted, during which the participants reviewed their respective unedited scenario video. The VRE interview included a question that required the participants to review their respective video and provide a running commentary (thinking aloud) with respect to their assessment, physiotherapy intervention and clinical decision-making processes. The ‘think aloud’ method [55] was integrated within the VRE interview schedule to encourage participants to independently review the video and verbalise their clinical decisions and clinical reasoning. The VRE interview video was generated using the QuickTime software (http://www.apple.com/quicktime/download/) screen recording feature. This enabled the voice recording and simultaneous capture of the screen displaying the participants’ simulation video. Data collection and storage was undertaken in line with the university ethical approval requirements.

**Table 1:** The integration of seven key elements underpinning the simulation design, development and analysis of the study

Elements	Details
1. Learner	Research study featuring final-year BSc (Hons) physiotherapy students from one university in the UK. All students undertook active roles within a uni-professional simulation scenario and debrief featuring a video-reflexive interview.
2. Facilitator	Facilitator and researcher roles were identified. Skills set established and formal training acquired within specialist areas of simulation scenario design, educational theory, debriefing, human factors and patient safety.
3. Theories and educational practices	The methodological design was informed by social constructivism [47, 48] and socio-material (complexity) theoretical perspectives [49–51, 60]. The scenario and video-reflexive interview embraced social constructivist theories including Vygotsky’s [70] zone of proximal development, situated and authentic learning [72]. Educational practices within the existing physiotherapy curriculum included blended learning [17], flipped classroom [71], scaffolding [73] with increasing levels of complexity of scenarios and the provision of opportunities for deliberate practice prior to practice (clinical) placements.
4. Learning design characteristics	Learning objectives were in line with a social constructivism principles [48]. The instructional medium included high (equipment, environmental and psychological)-fidelity simulation, featuring a human patient simulator. The modality was an immersive clinical simulation scenario featuring an acutely deteriorating medical in-patient. The simulation scenario has been outlined in Table 2. The instructional method included self-directed learning. A high degree of realism was achieved through the use of authentic artefacts (equipment and environment) and scenario design. Antecedent, reality, conceptual and cues incorporated into the scenario were included [74, 75]. Fiction cues were avoided, and responses to intervention were realistic in terms of physiological responses and timing. The scenario was designed to replicate the complexity of an emergency on-call physiotherapy situation and piloted to minimise cognitive overload [19–21].
5. Pre-brief and debrief	Pre-brief information was provided in advance of the study through the participant information sheet in respect to the focus, style format, duration and use of assistive technology and discussed in person on the day of the study. Information was also detailed relating to the debrief procedures in writing and discussed verbally during the pre-brief (format, style, anticipated duration and use of video-recording technology required to undertake the video-reflexive interview).
6. Linked learning activities	At the end of the video-reflexive interview (debrief), the linked learning activities were discussed with study participants. Participants were provided with a copy of their own video footage (scenario and video-reflexive interview), which they could combine with further written reflexive evidence for their personal e-portfolios. Further opportunities were available for the study participants to transform learning from the simulated scenario to practise during their forthcoming (final, elective) practice-based placement.
7. Outcomes	This study focused on exploring the experiences of pre-registration physiotherapy students’ experiences of managing a deteriorating simulated patient, the ability of the students to independently recognise errors, perceived elements of prior learning that may influence their performance and the value that pre-registration physiotherapy students attributed to the cardio-respiratory simulation-based learning experience. Video and thematic analysis was undertaken to explore knowledge, skills (technical and non-technical), attitudes, behaviours, clinical decisions and reasoning, elicited when managing an acutely deteriorating patient. A priori themes were integrated within the thematic video analysis from the acute illness management rubric [57] CSP framework [38] and non-technical skills for a surgeon’s observational behaviour tool [58].

**Table 2:** Summary of the emergency on-call physiotherapy scenario

The scenario exposes the pre-registration physiotherapy students to an adult medical patient whose condition has recently started to deteriorate. The patient was admitted to the Medical Ward via Accident and Emergency. An emergency on-call physiotherapy assessment is requested by the staff nurse.	
The learning objectives were to:	
• Demonstrate management of an acutely deteriorating medical in-patient	
• Implement appropriate physiotherapy intervention	
• Adhere to safe working practices including health and safety, moving and handling and infection control	
• Recognise universal precautions/unsafe practice and take appropriate action	
• Provide an structured handover	
Scaffolding: The scenario built on prior acute illness management and cardio-respiratory knowledge and skills embedded throughout the pre-registration physiotherapy curriculum. Antecedent cues included temporal (realistic physiological timing of responses to intervention), interpersonal cues (verbal prompts outlined in the simulated patient and healthcare assistant role profiles) and internal cues (manikin responses). Verbal, visual monitor display and written cues were provided to enable learners to discriminate conditions and prompt the desired consequence in a scenario (e.g. normalisation of physiological status in response to appropriate physiotherapy intervention). Participants were encouraged to ‘think aloud’ during the scenario.	
Role allocation and orientation: Randomization of participants to the role of the emergency on-call physiotherapists or healthcare assistant. All participants were then oriented to the simulation-based learning environment and equipment prior to the pre-brief.	
Pre-brief synopsis: Mr. Williams is a 61-year-old male who was admitted to the hospital 25 days ago. His admission diagnosis was multiple sclerosis and a recurrent urinary tract infection. The previous physiotherapy assessment findings indicate that he has low tone in his upper, lower limbs and thorax. He has restrictive thoracic movement in particular extension. Recommendations for moving and handling include using a slide sheet and hoisting from the bed to the chair or wheelchair. Assisted drinking is required and prompting Mr. Williams to cough post-swallow. The staff nurse reports that the patient is currently very tired, has a weak cough and has been sleepy since yesterday. He has become quite chesty since last night, when he had a drink of tea and thickened soup. An emergency on-call physiotherapy assessment is requested by the staff nurse.	
State 1 (initial assessment): The healthcare assistant is seated in the side room reviewing the patient’s notes. The patient’s physiological condition starts to deteriorate (in real time) as the physiotherapist enters the simulated side room. The physiotherapist is expected to complete an initial respiratory physiotherapy assessment.	
State 2 (physiotherapy intervention): The physiotherapist is expected to implement appropriate physiotherapy intervention based on clinically reasoned decisions. This included requesting a review and increase in oxygen therapy, repositioning the patient to optimise ventilation perfusion matching and selecting and administering appropriate chest physiotherapy intervention.	
State 3 (reassessment and handover): The physiotherapist is expected to reassess the patient’s status and provide a structured handover to the nurse/healthcare assistant.	

### Data analysis

Scenario and VRE interviews were transcribed verbatim (totaling 290 and 690 min, respectively). Qualitative thematic analysis of 12 scenarios and VRE interviews were undertaken using video analysis software and a thematic framework approach [56]. A priori themes were integrated within the thematic video analysis from the acute illness management (AIM) rubric [57] Chartered Society of Physiotherapy framework [38] and non-technical skills for a surgeon’s observational behaviour tool [58]. Quantitative descriptive statistics include the frequency and percentage of observed instances, e.g. events and technical and non-technical skills, present in relation to the scenario and interview analysis. For normally distributed data, the mean and standard deviation (SD) are reported. Otherwise, the median and inter-quartile range (IQR) are reported.

## Results and discussion

Twenty-one students (5 males and 16 females) participated in both the scenario and VRE interviews. The mean scenario and VRE interview durations were 24 min (SD = 5) and 57 min (SD = 10), respectively.

### The extent to which final-year physiotherapy students were able to independently manage an acutely deteriorating patient in a simulation context (Research question 1)

There are two focuses of analysis for research question 1: video analysis of physiotherapy skills, knowledge and behaviours (Table 3), non-technical skills (Table 4) observed during the scenario and thematic analysis of interview data (Table 5). Although all of the participants independently assessed the deteriorating simulated patient, their overall assessment was basic and in the majority of cases lacked structure. Despite some participants’ utilising the GMCCSI [54] acute illness management (ABCDE, airway, breathing, circulation, disability and exposure) approach to assess and manage the deteriorating simulated patient, this was not comprehensively completed by any of the participants. A median of 4.5 (IQR = 2–5) of the 19 assessment components and mean of 4.67 of the 10 management (SD = 1.07) were completed. The scenario pre-brief information did not specify that a particular approach to assessing the patient was required. This facilitated emergence, permitting multiple and diverse ways of thinking and acting on information provided within the scenario [59]. Within the VRE interview, participants also demonstrated attunement [51, 60] when openly discussing their assessment strategies and mental models and suggesting modifications to future practice (e.g. the adoption of a structured AIM approach to facilitate effective assessment and timely intervention). Attunement, emergence, disturbance and experimentation are some of the concepts within the complexity theory [51, 60] that have not previously been explored within physiotherapy SBE. During the scenario and VRE interview, participants demonstrated skills that aligned with the professional standards of physiotherapy practice [38] expected of entry-level physiotherapists (Table 3). Additionally, examples of how the participants demonstrated achievement of these physiotherapy standards relating to knowledge, skills, values and behaviours [38] within the VRE interview are also provided in Table 3. In the majority of cases, the findings mapped directly to the entry-level descriptors. There were some exceptions, where advanced graduate-level reflective practice descriptors [38] were observed during the VRE interview. All participants undertaking the role of the responding physiotherapist demonstrated a degree of competency in managing a deteriorating patient, which was characterised by their ability to prioritise actions, demonstrate and understand abnormal clinical findings and implement appropriate intervention [38, 61]. Participants demonstrated an ability to reflect-in-action [59] during the scenario and later review their own and others’ actions in the midst of uncertainty of the situation and physiological disturbances that unfolded during the scenario [51, 60].

**Table 3:** Demonstration of key physiotherapy knowledge, skills and behaviours

CSP framework domain [38]	Elicited through the scenario	Examples from the simulation scenario mapped to the graduate entry-level descriptors [38]	Elicited through the VRE interview	Examples from the video-reflexive ethnography interview mapped to the graduate entry-level descriptors [38]
Physiotherapy values	✓	Responsible for own actions, behaves ethically, undertakes an effective assessment	✓	Reflexive review of their own actions, behaviours and professionalism evident within the simulation scenario
Knowledge and understanding of physiotherapy	✓	Practice within complex generally predictable conditions which required the application of current physiotherapy knowledge	✓	Reflexive review of their own knowledge relating to the management of an acutely ill patient
Self-awareness	✓	Reflection-in-action of the limitation of knowledge and skills. Requesting help from an appropriate member of the multi-disciplinary team	✓	Demonstration of self-awareness during the reflexive review of personal practice, incorporating feedback from others to identify and articulate their personal values, ways of working, then analysing how these may influence their behaviour and practice
Physiotherapy practice skills	✓	Assessment and management of the acutely deteriorating patient including the modification of techniques in response to patient feedback and physiological changes in the patient’s condition Process and critically analyse information in complex and predictable situations where data/information comes from a range of sources or is incomplete	✓	Reflexive review of physiotherapy and generic AIM skills in the management of an acutely deteriorating patient. Demonstration of the ability to evaluate their own and others’ performance. By reflecting on clinical decisions and evaluating the outcome of intervention and the overall scenario, participants recognised this may inform their future practice (advanced graduate level)
Communicating	✓	Demonstration of sharing information, advice and ideas with others using a variety of media (including spoken, non-verbal, written). Modification of communication to meet individuals’ preferences and needs	✓	Evidence of self-awareness and ability to modify their communication in response to feedback (e.g. from the patient and peer) to meet the needs of others involved in the simulation scenario
Promoting integration and teamwork	✓	Demonstration of the ability to work effectively with others to meet the responsibilities of professional practice	✓	Reflexive review of their own practice within the scenario including working effectively with others to meet the responsibilities of professional practice and identifying situations where collaborative approaches could add value to practice and improve patient safety (in particular moving and handling and infection control)
Helping others learn and develop	x		✓	Demonstration of self-awareness of learning preferences and started to independently identify some personal learning and development needs relating to assessment and physiotherapy intervention options (advanced graduate level)
Managing self and others	✓	Actively takes some responsibility for the work of others (e.g. delegation of tasks within the scenario) Modification of personal behaviour and actions in response to peer/patient feedback to meet the demands of the situation	✓	Reflexive review of the ability to take some responsibility for the work of others (e.g. delegation of tasks within the scenario). Demonstration of an ability to suggest modification of their personal behaviour and actions in response to peer feedback, to meet the demands of similar situation in the future, in order to enhance own performance
Putting the person at the centre of practice	✓	Demonstration of respect for the HCA and simulated patient by acknowledging their unique needs, preferences and values, autonomy and independence in accordance with legislation, policies, procedures and best practice	✓	Acknowledging the unique needs and preferences of the patient and peer in accordance with legislation (e.g. moving and handling or infection control policies, procedures and best practice)
Respecting and promoting diversity	✓	Demonstration of respect for the ability to work constructively with people of all backgrounds and orientations by recognising and responding to individuals’ expressed beliefs, preferences and choices	✓	Reflexively reviewed their own practice within the scenario including working constructively with others (physiotherapist, HCA, patient) and recognising and responding to individuals’ expressed beliefs, preferences and choices (e.g. treatment preferences and subjective comments relating to fatigue or requiring a rest from treatment)
Ensuring quality	✓	Recognised situations where the effectiveness and efficiency of intervention are compromised and take appropriate action	✓	Reflected on personal performance and with guidance, projects that this evaluation can be used to enhance the effectiveness, efficiency and quality of future practice (advanced graduate level)
Lifelong learning	✓	Identified knowledge/skill deficits, request assistance and identify further personal development requirements (in particular relating to physiotherapy intervention and suction)	✓	Assessed own personal learning and development needs and preferences. Reflected on the learning process
Practise decision-making	✓	Effective use of a wide range of routine and some specialised approaches (AIM) and techniques to systematically collect information from a variety of sources relevant to the situation	✓	Reflexively reviewed the effectiveness of a routine and specialised AIM approach and techniques to systematically collect information from a variety of sources relevant to the situation

*VRE* video-reflexive ethnography, *HCA* healthcare assistant, *AIM* acute illness management

**Table 4:** Video analysis of physiotherapy non-technical skills observed during the scenario

Theme	Subtheme	Definition	Frequency/12 scenarios
1) Situational awareness	1.1 Gathering information^a,^ ^b^	Uses the patient’s medical records, charts, and x-ray to ascertain the pertinent information	12
	1.2 Understanding information^a,^ ^b^	Verbalises awareness of the situation and evolving physiological status of the patient	12
	1.3 Projection^a^b	Demonstrates an awareness of possible future states (e.g. changes in the physiological states of the patient)	5
	1.4 Anticipating future states^a,^ ^b^	Anticipates possible future states (e.g. changes in the physiological states of the patient)	3
2) Decision-making	2.1 Considering options^a,^ ^b^	Verbalises assessment/interventions/management options relevant to the patient or situation	12
	2.2 Selecting and communicating options^a^b	Selects and communicates options relevant to the patient or situation	12
	2.3 Implementing decisions^a,^ ^b^	Implements decisions appropriately	12
	2.4 Reviewing decisions^a,^ ^b^	Reviews decisions following implementation of intervention or during the handover	11
3) Task management	3.1 Planning and preparing^a,^ ^b^ 3.2 Flexibility or responding to change^a,^ ^b^	Appropriately prepares the environment before implementing intervention Adopts a flexible approach to assessment of the patient, responding to changes in the patient’s needs	11 7
4) Leadership	4.1 Setting standards^a,^ ^b^	Demonstrates an awareness of moving and handling/infection control procedure	11
	4.2 Maintaining standards^a,^ ^b^	Adheres to moving and handling policy standards. Adheres to infection control policy in relation to the management of the patient. Raises the awareness of the need for infection control equipment	7
	4.3 Supporting others^a,^ ^b^	Demonstrates supportive attitude towards others in their role/duties/actions relating to the assessment/treatment intervention	2
5) Communication and teamwork	5.1 Exchanging information^a,^ ^b^	Demonstrates the ability to exchange verbal/written information with others	12
	5.2 Establishing a shared understanding^a,^ ^b^	Demonstrates the ability to communicate information to ensure a shared understanding amongst members of the team (e.g. present or via telephone conversation) regarding the patient’s current/evolving status	7
	5.3 Co-ordaining team activities^a,^ ^b^	Demonstrates the ability to coordinate team activities (e.g. undertaking the lead role in moving and handling, repositioning the patient, suction)	10
	5.4 Communicating requirements^a,^ ^b^	Demonstrates an ability to communicate requirements (e.g. requesting further assistance from other members of the multi-disciplinary team)	10
	5.5 Use of a standardised communication tool	Uses a standardised communication tool (e.g. SBAR) when communicating with other members of the multi-disciplinary team	1

*SBAR* Situation, Background, Assessment and Recommendation [62]

^a^A priori basic themes from the Non-Technical Skills for Surgeons (NOTSS) behaviour rating tool [58]

^b^CSP behaviours, values, knowledge and skills framework [38]

**Table 5:** Thematic analysis—themes 1 to 4

Theme 1—behaviour	
Subtheme	Examples
1.1 Professionalism	• I feel like I am really loud and might be a bit condescending to be so loud, like the patient is deaf. Yeah, because I always listen to my voice and I am thinking why was I so loud, he can hear me…it’s something that subconsciously I have started doing when I talk to patients and it’s something that I need to tone down.
1.2 Situational awareness	• So I went to listen to his chest, noticed the monitor going off, it was the sats (referring to oxygen saturations) dropping but I think they just dropped to 89/88, something like that so I was hoping it was a bit of a drop and he would pick up on his own. But as I started auscultating the saturations continued to drop so I stopped auscultating, increased his oxygen because my main concern was to keep his sats up. Whereas if they dropped too low things could start deteriorating more quickly, so if we get his sats up to a reasonable level and they stay there we could continue with the assessment and find out a little bit more about it. That’s when I called (referring to the healthcare assistant) over to help me just reposition him and see if it was just a matter of positioning, that his sats were dropping. And then, I think as we go on I finally reposition him and he doesn’t pick up quick enough for my liking, so we upped the oxygen.
1.3 Communication	• That was me jumping in then, there when I should have stepped back. Sorry. I am vocal too, so it was a bit of a clash because I should have just let you finish talking but you know how it is. It’s hard we are both, both thinking the same thing. • We are speaking amongst ourselves rather than speaking to him. I think he kept asking us questions, which is good, and I think I started talking a bit more towards him.
1.4 Knowledge and skill deficit	• We tried ringing for help but I think if I did that in an actual clinical setting I would feel a bit daft having to ring for the nurse to come and help sort the humidification out. I was a bit confused with it. • I wasn’t sure whether to use the non-rebreathe mask or the 60 % venturi mask. I was like ask the healthcare assistant…That’s why I hesitated, because I was unsure what to do. Brain freeze there.
1.5 Clinical reasoning	• So I also wanted to get him more of a high sitting position because in that slumped position he would be able to breathe more effectively, so to increase his V/Q (referring to ventilation perfusion) matching. I tried to use the sliding sheet to do that.
1.6 Error identification	• And then, we are just putting our gloves and things on here, which I should have done at the start but I am just doing that now. • At this point I didn’t have gloves or an apron on, I should have. I still hadn’t introduced myself after 50 seconds. Throughout all the assessment, I was being quite slow to get the gloves on and should have been quicker.
Theme 2—independent error identification	
Subtheme	Examples
2.1 No errors	• I don’t think I did anything majorly wrong. Like I said the main thing I would have probably, would have left him on his other side. If I did do anything wrong I don’t think it was anything that would have put him any major danger or risk. But as far as I can tell I didn’t do anything that I didn’t clinically reason to be safe and in the patient’s best interest. • I don’t think so, I think you mentioned about the nebulisers, I don’t know if I would call it a clinical error or not but obviously it would help with the moving the secretions so probably shouldn’t have done suctions straight away.
2.2 Assessment	• I wasn’t too sure what I was hearing with the crackles…So if I did it again I would probably try to clinically reason it a bit better so that I wouldn’t make errors like that.
2.3 Communication	• I think I would have hopefully done better with the telephone conversation to the nurse to explain what I had done and how Levi was.
2.4 Infection control	• Also just things like putting my gloves and aprons on and just simple things like that I forgot to do which maybe I wouldn’t have forgotten to do in a real hospital setting. I would have done that automatically… although that is quite real, it is real patients and I just think about it more when I am in that setting. It just seems to come more naturally to me to do those things. Because it’s a real person they might have real infections, I think it makes you more aware to it.
2.5 Manual handling	• I think at one point I did lose control of his head when lifting him up. I would ensure that didn’t happen but I did ensure that didn’t happen afterwards. • We should have probably put the bed flat as well before we moved him so we were kind of going uphill which made it a lot harder
2.6 Intervention	• Well giving him oxygen without asking for a prescription from a doctor that’s a major error. • When she rolled on the right hand side I kind of mumbled good lung down hoping that she would go towards me. And I grumbled when she rolled towards me, try towards me. But yeah, I think that was one of the errors.
Theme 3—prior experience	
Subtheme	Examples
3.1 University units	• …Something like doing this would have been helpful in uni but anything that we have done has been nothing so life-like, so I don’t think it has prepared me. • …my work at uni gave me the background knowledge for assessments and treatment interventions. Then clinical placements helped build on that but I hadn’t done the critical care placement. • …in uni, you’re just doing it on your peers so you don’t think about it as a real patient and deteriorating and you don’t have that pressure on you so I don’t think that’s really prepared you for that kind of situation. • …Something like doing this would have been helpful in uni but anything that we have done has been nothing so life-like this so I don’t think it has prepared me.
3.2 Placement	• I think my clinical placement more so prepared me for it because then, I did a lot of assessments. So, I can visualise assessments and treatments so I drew on those. • No. No, I don’t because on placement I had done a placement on ICU (Intensive Care Unit). Well partly on ICU but it was a surgical ICU, so people were only there who had major surgery, they weren’t actually poorly as such so I haven’t had experience with people actually deteriorating on me.
3.3 Acute illness management (AIM) course [57]	• …when I did the AIM course through uni, I think this helped me understand what to do in a situation like this. • Even though AIM was a whilst ago, going over that AIM thing constantly it’s kind of in my head…it’s good to know that in my head somewhere, it’s there.
Theme 4—value of simulation and reflexivity	
Subtheme	Examples
4.1 Skills development	• I think so, definitely because it gives you a chance to put the theory into practice without the pressure of it being an actual patient. So, if you go wrong then you can remedy it without feeling bad or worrying about what your educator thinks of you.
4.2 Increased self-awareness	• I think it will definitely help me on my elective because I will be doing respiratory, so I might not feel quite so daunted coming to see someone that is acutely ill. I think it’s quite good as well watching back yourself on a video you don’t realise at the time how you come across and how long time seems, when sometimes it feels like its flying but really it’s just not. I think it’s just helpful to get an overall picture of you and then reflecting on that as well.
4.3 Placement preparation	• Should I have done this before I went on my ICU placement I wouldn’t have been so overwhelmed when seeing the patients so acutely ill and also when I first went on that placement. I was completely scared but obviously from that scenario that’s what happens in ICU so should I have done this before, I would have been a lot less nervous so more prepared. • Yeah, it’s like a refresher because it’s been six months now since the last placement and its only three weeks until we go again…I am on orthopaedics, there are not so many nurses, so I am more nervous when I treat them. And I would be able to spot the signs now.
4.4 Added realism	• The exposure to the pressure I think it’s a good realistic thing that you wouldn’t get in a skills scenario like [name] said, with the beeping with somebody actually realistically in front of you who is acutely unwell it’s definitely a beneficial thing to be exposed to. • I feel there is massive benefit to undergraduates and pre-reg experience as it did replicate a clinical environment…and I personally felt that I learnt more about aspects of treatment and assessment rather than undertaking a less realistic assessment on one of your colleagues in university as a student.
4.5 Patient safety	• I think it will massively impact on patient safety through continually being to be able to adapt new environments even for the same patient, where many problems could be presented. For example the patient we saw today, a completely different problem could be shown with the same dummy allowing a person to experience all various different types of problems that would present in clinical practice with real patients. Therefore having all these learning experiences to draw from that they have reflected on and thought out loud about would definitely improve their clinical practice with real patients like quality of care and safety. • …you’re allowed to make mistakes, like the mistake that I made and it’s not going to matter or it’s not going to impact the patient’s safety in this environment anyway and you definitely, I know I do, learn by doing and therefore learn by making mistakes as well. So to have made those mistakes in a safe environment you then go out and don’t make the same mistakes out there when it is going to potentially affect the patient’s life. So definitely.
4.6 Video review	• I think the video review is definitely going to have helped because, whilst I was in there it felt like a train wreck but having come out and being able to talk about it and think about it. It helps to recognise where you went wrong, because I think if I hadn’t done this I would have gone away and just thought that was a disaster and tried not to think about it as much as I could. So, I definitely think that’s going to have helped.
4.7 Digital video disc (DVD)	• …with the DVD, if I watch it I might be able to see more things that could have been different or better or that were good so I think it will help. • In reference to general continuous professional development, I am going to complete a written reflection and also I feel the DVD I will continually revisit that so I have got a constant picture of how next time I can always improve my respiratory assessment and treatment.

Table 4 provides a summary of the participants’ non-technical skills, which mapped to the CSP behaviour, value, knowledge and skill framework [38] and non-technical skills for surgeons [58].

Participants demonstrated variable situational awareness skills (Table 4, theme 1). All participants demonstrated an ability to gather appropriate information and demonstrated an immediate understanding of the situation (Table 4, theme 1). To a lesser extent, participants were able to project or anticipate possible future changes in the patient’s condition. All participants verbalised their decisions and selected and communicated their options, implementing them appropriately, and 11 reviewed their decisions (Table 4, theme 2). In the majority of scenarios, participant demonstrated their ability to independently manage tasks, including planning and preparing the environment and equipment, demonstrating and awareness of standards, responding flexibly to changes in the patient’s verbalised needs or physiological parameters (Table 4, theme 3). Leadership skills varied amongst participants, in particular relating to setting and maintaining standards for moving and handling and infection control (Table 4, theme 4). Only two participants demonstrated a supportive attitude towards the healthcare assistant during the assessment or intervention (Table 4, theme 4). Communication and teamwork skills (Table 4, theme 5) also varied across the scenarios. An additional subtheme was identified relating to the teamwork and communication theme 5, which referred to the use of a standardised communication tool [62] when communicating with other members of the multi-disciplinary team (Table 4, subtheme 5.5). During the scenario and VRE review, participants discussed their own level of expertise, requested help and delegated tasks appropriately. Overall, participants demonstrated attunement through their ability to listen to the patient and HCA and patient, observing, touching and sensing the scenario that was unfolding [51, 60]. This study has provided a unique exploration of physiotherapy non-technical skills demonstrated within a simulation scenario. Findings indicate that scenario and debriefing learning outcomes focusing on non-technical skills could be factored into the physiotherapy curriculum to enhance patient safety.

During the VRE interview, participants independently reviewed their unedited simulation video. As participants reviewed their respective video of their scenario, they reflexively discussed their behaviour. The thematic analysis is presented in Table 5, theme 1: behaviour. Six themes were identified including: professionalism, situational awareness, communication, knowledge and skill deficit, clinical reasoning and error identification. Participants discussed their professional behaviour and future modifications (subtheme 1). Fluctuations in the simulated patient’s physiological status and how these affected the situational awareness, clinical reasoning and choice of intervention were discussed (subtheme 2). The impact of communication with each other and the patient was recognised by participants (subtheme 3). Knowledge and skill deficits related to respiratory physiotherapy and oxygen therapy intervention (subtheme 4). Participants reviewed their ability to clinically reason their actions (subtheme 5) and identify errors during the scenario (subtheme 6). The scenario was designed to replicate the complex interactions a respiratory physiotherapist undertakes to function effectively within their clinical environment, including constant observation of the patient and the noise and visual disturbances generated by monitors and equipment located within the visual field around the patient’s bed space and ward [45, 49, 51, 60]. Participants discussed their interaction with the environment, artefacts embedded in the scenario and their resultant behaviours [51, 60]. Despite being pre-registration physiotherapy students, the participants in this study demonstrated an ability to recognise the complexities and dynamics that unfolded within the simulated scenario and to suggest alterative practices in future situations [52, 54].

### The extent to which final-year physiotherapy students were able to independently recognise errors within a simulation-based learning experience (Research question 2)

There are two focuses of analysis for research question 2. Table 6 presents the video analysis of the simulation scenarios, identifying error types and defences utilised by participants to mitigate errors. Additionally, thematic analysis of the participants’ ability to recognise errors is presented in Table 5, theme 2.

**Table 6:** Video analysis of error types and defences

Theme	Subtheme	Definition	Frequency
1) Latent error^a^	1.1 Multiple oxygen therapy policies	• Presence of multiple oxygen therapy polices	12
2) Active failures^a^	2.1 Coordination^b^	• Error during discussion with the patient • Incomplete/incorrect readback/feedback	2 4
	2.2 Verification^b^	• Error related to the identification of the patient • Failure to verify the infection control status	3 1
	2.3 Monitoring^b^ 2.4 Intervention^b^	• Partially completes a respiratory assessment • Failure to recognise abnormal assessment findings • Administers incorrect/ineffective physiotherapy intervention • Error in physiotherapy skill administration • Failure to obtain oxygen prescription prior to administration • Infection control violation • Moving and handling violation	11 5 7 1 9 3 7
3) Error-producing factors^a^	3.1 Environmental 3.2 Individual	• Lack of environmental provisions • Lack of knowledge • Lack of physiotherapy skills	12 10 20
4) Defences^a^	4.1 Identifies the patient 4.2 Effective communication with the patient 4.3 Effective communication/complete readback 4.4 Recognises abnormal assessment findings 4.5 Seeks/obtains oxygen therapy prescription 4.6 Correctly implements oxygen therapy 4.7 Appropriate chest physiotherapy intervention 4.8 Adheres to infection control procedures 4.9 Adheres to moving and handling procedures 4.10 Structured handover 4.11 Unstructured handover	• Correct identification of the patient • Demonstrates effective communication with the patient • Demonstrates effective communication/complete readback/feedback • Demonstrates the ability to recognises abnormal assessment findings • Seeks/obtains oxygen therapy prescription from doctor prior to administration • Correctly implements and increase in oxygen therapy • Selects and delivers appropriate chest physiotherapy intervention • Demonstrates adherence to infection control procedures • Demonstrates adherence to moving and handling procedures • Structured handover using SBAR tool • Handover (unstructured) Total	2 8 4 8 3 12 7 11 5 1 10 107
Errors corrected by participants following reassessment of the patient (reflecting-in-action during the scenario)			2
Errors uncorrected by participants (during the scenario)			105
Errors identified during reflexive interview (reflecting-on-action during the video-reflexive interview)			26
Total errors identified (during the scenario and interview)			28
Total unidentified errors (during the scenario and interview)			79

*MRSA* methicillin-resistant staphylococcus aureus, *SBAR* Situation, Background, Assessment and Recommendation [62]

^a^A priori error typology themes from Reason [76]

^b^Active failure a priori subthemes from Henneman et al. [63]

Video analysis identified 107 errors during the scenario (Table 6).

Participants’ independently identified 28 of 107 these errors, two during the scenario and the remaining 26 during the unedited review of their scenario during the VRE interview. Thus, 79 errors (74 %) were unrecognised by the participants either in-action (during the scenario) or on-action (when reviewing their video). The unrecognised errors related to key physiotherapy skills (poor auscultation skills, suction skills, failure to recognise abnormal assessment findings, failure to seek/obtain the prescription for the change in oxygen therapy prior to administration, errors in the delivery of physiotherapy intervention and a communication error). The participants’ limited ability to independently recognise errors encountered in their own scenario was consistent with a previous study involving pre-registration nursing students [63]. Thematic analysis of the VRE interview identified six themes in relation to the participants’ ability to independently identify errors in their own scenario including no errors, assessment, communication, infection control, moving and handling and intervention. No previous studies have explored pre-registration physiotherapy students’ ability to identify errors in their simulated practice using VRE. During the interview, the participants lacked insight into their own abilities regarding cardio-respiratory physiotherapy and AIM skills, moving and handling, and infection control procedures (Table 5, theme 2). This is an important finding since a lack of insight into one’s own skills can have a fundamental impact on patient safety [64]. This study has identified that the use of VRE has the potential to facilitate the identification of participants who lack insight into their knowledge, skills and behaviours and has the potential to play an important part in improving patient safety [52]. Ahmed et al. [65] proposed that reflection on personal performance and errors is critical in ensuring deep learning and positive behavioural change.

### Perceived elements of prior learning that may influence performance within a simulation-based learning experience (Research question 3)

Table 5 (theme 3) presents the thematic analysis of the VRE interview data relating to the influence of prior learning on performance within the simulation-based learning experience. Three subthemes were identified: university units, placement and the AIM course [57]. During the VRE interview, participants shared the impact of personal experiences, which they perceived may have been central to their actions, clinical decisions and physiotherapy practice (Table 5, theme 3). Whilst participants reported that the university units had influenced their actions, clinical decisions and intervention provided during the scenario, they indicated that this was a unique, realistic experience that had not been previously provided within cardio-respiratory skill sessions (subtheme 1). Individual placement experiences reportedly varied with some participants organising a forthcoming respiratory-biased elective, as they had not yet been responsible for the management of an acute or critically ill patient during placements (subtheme 2). Whilst the participants perceived the AIM course (subtheme 3) provided the underpinning knowledge and skills to enable them to grasp the key concepts of the scenario (identification of the deteriorating patient, identification of abnormal clinical features and initiation of appropriate intervention), none of the participants fully completed an AIM assessment (including airway, breathing, circulation, disability and exposure components) during the scenario (research question 1).

### Perceived value attributed to the cardio-respiratory simulation-based learning experience (Research question 4)

Table 5 (theme 4) presents the thematic analysis of the VRE interview data relating to the perceived value attributed to the cardio-respiratory simulation-based learning experience. Seven subthemes were identified: skill development, increased self-awareness, placement preparation, added realism, patient safety, video review and digital video disc. Participants felt that the experience provided an opportunity to further develop their skills, putting theory into practice (subtheme 1) and increasing their self-awareness (subtheme 2). The experience was deemed valuable for placement preparation or as a refresher (subtheme 3). In particular, the participants valued the realism afforded by the scenario (subtheme 4) and the varied opportunities that SBE provides in relation to positively impacting on patient safety (subtheme 5). Value was reportedly attributed to the scenario, as it provided an opportunity to practise and utilise physiotherapy skills in a safe environment, learning from their own mistakes, without risks to patients. Participants also valued the opportunity to reflexively review their simulation video to influence future practice (subtheme 6), which afforded the ability to scrutinise their own and each other’s behaviour [52]. Additionally, they valued the opportunity to extrapolate their existing behaviours and activities within the simulated scenario and project into the near future (elective placement, EOC situations and postgraduation). The possibility to develop action plans and use the digital simulation resources (generated from the scenario and reflexive review) to evidence their personal development, within their electronic portfolio [66, 67], was positively reported. Value was also attributed to the opportunity to repeatedly reflect on their experience using the digital resources provided in preparation of future learning activities in their forthcoming placement (subtheme 7), which concurs with the literature [66, 67]. The scenario and VRE interview permitted experimentation of knowledge, skills, clinical reasoning and decision-making within a situation that was deemed by participants to be a realistic representation of acute respiratory physiotherapy practice and valuable for pre-registration physiotherapy students prior to placements [51, 60]. Participants valued the opportunity to influence future practice, during the video-reflexive review of their scenario, which afforded the ability to scrutinise their own and each other’s behaviour [52, 54]. These are essential skills required for autonomous practice as a physiotherapist [36–38].

### Summary of the research findings

Participants independently assessed the patient within a simulation context, but their overall assessment was basic and in the majority of cases lacked structure and depth. During the scenario, participants worked within the expected professional standards of physiotherapy practice expected of entry-level physiotherapists [38]. Participants demonstrated an ability to reflect-in-action (during the scenario) in the midst of uncertainty of the situation and physiological disturbances that unfolded during the scenario and later reviewed their own and others’ actions during the VRE interview [59]. However, they demonstrated a limited ability to independently recognise errors in their own simulated practice. Latent errors, active failures (communication verification, monitoring and intervention), error-producing factors and a series of effective defences to mitigate errors were identified through video analysis. Perceived influential factors affecting performance within the simulation scenario related to learning attributed to the physiotherapy academic and placement components and participation in an additional, voluntary AIM course. The perceived value of the simulation scenario was enhanced by the opportunity to reflexively review their own simulation video and the realism afforded by the scenario design.

One additional product of this study was the development of an ‘integrated simulation and technology-enhanced learning’ (ISTEL) framework. The ISTEL framework integrates the theoretical and educational practices that underpinned the simulation design, development and analysis of the study, and implementation and evaluation of STEL interventions (Additional file 1). In this instance, STEL is defined as the inclusion of simulation, simulated patients, and other ‘innovative educational technologies, such as e-learning, smart phones, which provide unprecedented opportunities for health and social care students, trainees and staff to acquire, develop and maintain the essential knowledge, skill, values and behaviours needed for safe and effective patient care’ [17:6]. It incorporates technology to enhance learning such as video-recording equipment to support the use of video debriefing, video reflexivity and generation of podcasts of scenarios. Further details of the development of the ISTEL framework and how it can be used to support the design, development, implementation and evaluation/research of STEL will be presented elsewhere.

### Methodological strengths and limitations

This study highlighted the power of video reflexivity to explore and uncover the multiple and complex realities of managing an acutely deteriorating patient in a simulation context, which are constructed via social, verbal and non-verbal interactions with the patient, others and the environment [49–51, 53, 60]. This study demonstrated that the VRE was successfully employed to facilitate error recognition and patient safety awareness. It allowed the participants to question their own knowledge, skills and behaviours in a manner that impacts on themselves and how they relate to patients in a simulated learning environment [54]. The visualisation and narratives provided by the participants during the VRE interview offered the ability to understand the complexity of learning within a simulation context. Findings of this research provide valuable insights to inform future VRE research regarding physiotherapy practice, integration of educational methods to augment patient safety awareness and participant-led innovations in safe healthcare practice. Carefully designed and executed STEL experiences, coupled with video-reflexive methods, can offer a safe learning environment to allow learners to explore routine, evolving and complex clinical situations whilst allowing them to learn to be become comfortable with making and exploring errors (mistakes/violations).

Reassuringly, findings have indicated the participants worked within the expected professional standards of physiotherapy practice [38]. The use of VRE allowed participants to openly reflect on their knowledge, skills, attitudes and behaviours as well as identify errors and develop appropriate remedial action. This study demonstrated that VRE methods were successfully employed to explore the management of a deteriorating patient and facilitate error recognition and patient safety awareness, which may be equally beneficial to exploring medicine, nursing and allied health profession education and practice. It allowed the participants to question their own knowledge, skills and behaviours in a manner that impacts on themselves and how they relate to patients in a simulated learning environment [54].

This study highlights that learning is highly complex, requires context and continually evolves through social interaction [51, 60], which may be extrapolated to medicine, nursing and allied health professions involved in managing deteriorating patients. One strength of this study is gained through the pragmatic approach, which drew on both qualitative and quantitative approaches, to explore learning and practice within the simulated environment and to establish the extent to which transformations in learning and/or patient care are realised, or not, by the learner [68]. By employing and triangulating qualitative and quantitative approaches to explore multiple levels of impact, the complexities of learning can be explored identifying areas of best practice and helping to remedy any deficits, to enhance the transformation between theory and practice [68].

The authors acknowledge the potential influences an insider-researcher perspective may have on this study. Whilst insiders-researchers have the potential to facilitate a greater understanding of the participants’ (physiotherapy) practices and social interaction, we also acknowledge the potential effect of acquiescence, owing to the principal investigator’s role as an academic on the physiotherapy programme [53, 69]. Additionally, the authors acknowledge that being an insider-researcher also brings various disadvantages, including the potential loss of objectivity due to relative familiarity of physiotherapy practice and introduction of bias through incorrect assumptions based on the researchers’ prior knowledge [69].

We acknowledge that the findings of this study are drawn from a BSc (Hons) physiotherapy programme, from one HEI in the UK. The participants were also only exposed to one deteriorating adult patient scenario. An adult scenario was selected as pre-registration students have limited exposure to paediatric patients. Participants were not followed up after their elective or graduation. It is acknowledged that such follow-up may have provided a valuable opportunity to explore the transfer of skills to their elective and employment beyond graduation.

### Recommendations for education and research

Findings from this study have influenced ongoing scenario design within the physiotherapy curricula in relation to further embrace the complexity of learning of learning within simulation-based learning activities. Whilst the scenario embraced the complexity of emergency on-call situations, the findings have indicated that it is important to recognise the knowledge and skills (technical and non-technical) limitations of pre-registration physiotherapists. In this instance, the scenario was designed to replicate an on-call situation that the participants may encounter within the forthcoming months, as a qualified physiotherapist. The combination of SBE and VRE can provide supportive learning opportunities that enable learners to move from their current level of knowledge and understanding, into what is referred to by Vygotsky as the ‘zone of proximal development’ [70].

In this study, participation in the scenario and VRE promoted the use of problem-solving skills, as participants drew on their current and pre-existing knowledge gained from the academic and placement experiences, to select appropriate information, construct hypotheses and make appropriate clinical decisions. However, the findings indicated that repetitive practical reinforcement of essential physiotherapy clinical skills (e.g. auscultation, positioning, moving and handling and infection control procedures) and non-technical skills would be of benefit to pre-registration physiotherapy students participating in scenarios. Additionally, overt scaffolding of non-technical skills (e.g. situational awareness, decision-making, task management, communication and teamwork and leadership) may help to improve patient safety within scenarios and facilitate translation through practice. The provision of additional ‘flipped classroom’ [71] resources (educational videos and podcasts) have been introduced to support repetitive practice of essential technical and non-technical skills required to manage cardio-respiratory and acutely deteriorating patients.

## Conclusions

In summary, this study has presented a unique insight into the experiences, skills, attitudes, behaviours and error recognition abilities of pre-registration physiotherapy students managing an acutely deteriorating patient in a simulation context. This study has demonstrated that the combination of SBE and video-reflexive methods has the potential to facilitate the identification of participants who lack insight into their knowledge, skills and behaviours and has the potential to play an important part in improving patient safety. SBE and VRE could be employed to explore the complexities of healthcare professional learning and practice beyond cardio-respiratory, in particular to highlight key gaps in the curricula, as well as the existence/deficits in learner knowledge, skills and behaviours. Findings of this research provide valuable insights to inform physiotherapy practice, integration of educational methods to augment patient safety awareness and participant-led innovations in safe healthcare practice.

## Ethics approval and consent to publish

Ethical approval was granted by the Faculty of Health, Psychology and Social Care at Manchester Metropolitan University (reference number 1102). Participant consent was obtained to publish findings from the scenario and VRE interview data.

## Additional file

Additional file 1: The Integrated Simulation and Technology Enhanced Learning (ISTEL) Framework. The ISTEL framework was developed by synthesizing the literature focusing on the theoretical perspectives and educational practices that inform the preparation, intervention and research or evaluation of STEL, with the methodological design and analysis of this study. (PDF 4325 kb)

## Abbreviations

HCA: healthcare assistant
ISTEL: integrated simulation- and technology-enhanced learning
SBE: simulation-based education
STEL: simulation- and technology-enhanced learning
VRE: video-reflexive ethnography
