# Supplementary material for: Using video-reflexive ethnography and simulation-based education to explore patient management and error recognition by pre-registration physiotherapists
Source: Adv Simul (Lond). 2016 Mar 22;1:9. doi: 10.1186/s41077-016-0010-5 (PMC5806348; doi:10.1186/s41077-016-0010-5)
Supplement: Supplementary file 1 — The Integrated Simulation and Technology Enhanced Learning (ISTEL) Framework. The ISTEL framework was developed by synthesizing the literature focusing on the theoretical perspectives and educational practices that inform the preparation, intervention and research or evaluation of STEL, with the methodological design and analysis of this study. (PDF 4325 kb) [file 41077_2016_10_MOESM1_ESM.pdf]

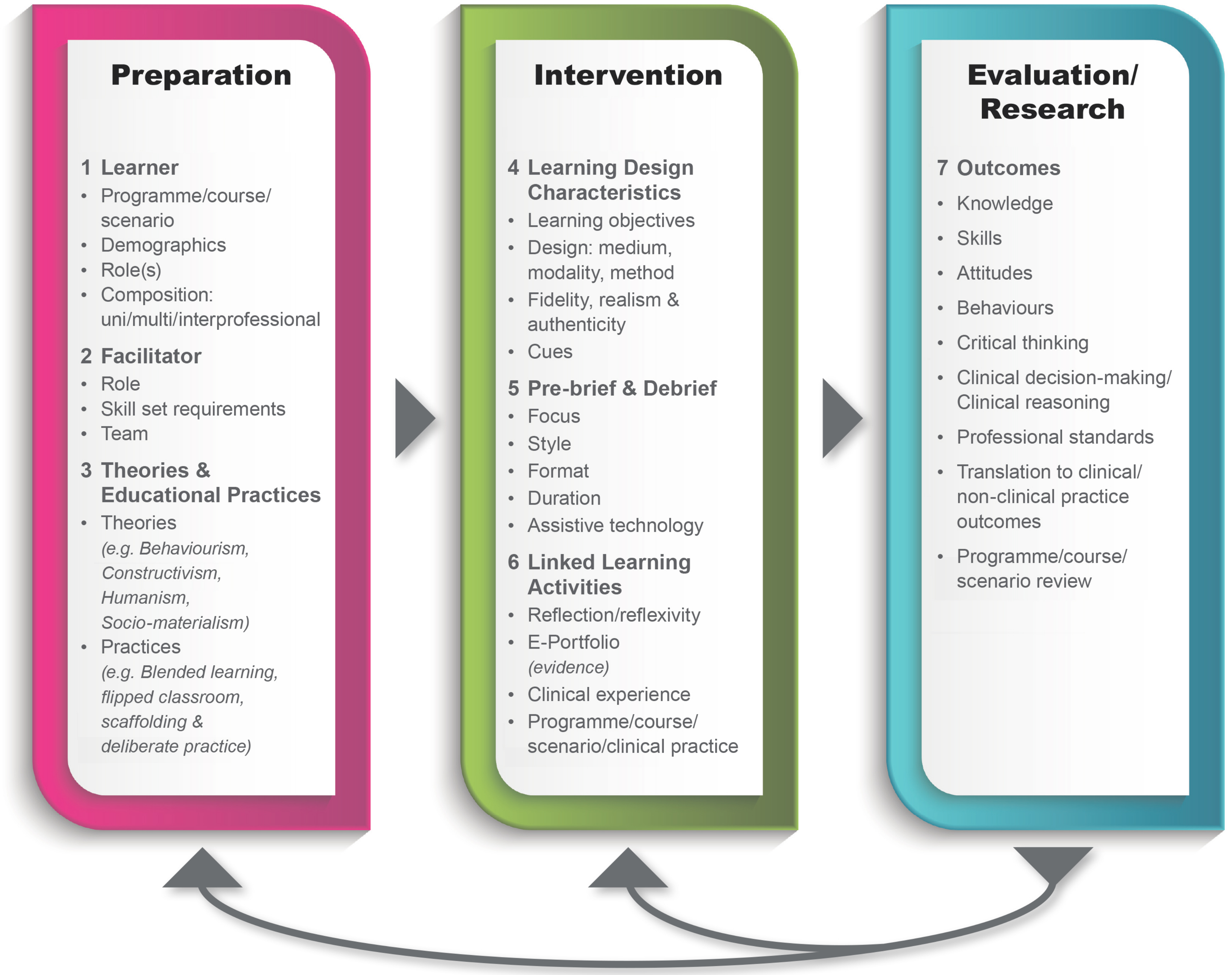
Preparation
1 Learner
• Programme/course/ scenario
• Demographics
• Role(s)
• Composition: uni/multi/interprofessional
2 Facilitator
• Role
• Skill set requirements
• Team
3 Theories & Educational Practices
• Theories (e.g. Behaviourism, Constructivism, Humanism, Socio-materialism)
• Practices (e.g. Blended learning, flipped classroom, scaffolding & deliberate practice)
Intervention
4 Learning Design Characteristics
• Learning objectives
• Design: medium, modality, method
• Fidelity, realism & authenticity
• Cues
5 Pre-brief & Debrief
• Focus
• Style
• Format
• Duration
• Assistive technology
6 Linked Learning Activities
• Reflection/reflexivity
• E-Portfolio (evidence)
• Clinical experience
• Programme/course/ scenario/clinical practice
Evaluation/ Research
7 Outcomes
• Knowledge
• Skills
• Attitudes
• Behaviours
• Critical thinking
• Clinical decision-making/ Clinical reasoning
• Professional standards
• Translation to clinical/ non-clinical practice outcomes
• Programme/course/ scenario review
